# Lasting Effects of Workplace Strength Training for Neck/Shoulder/Arm Pain among Laboratory Technicians: Natural Experiment with 3-Year Follow-Up

**DOI:** 10.1155/2014/845851

**Published:** 2014-03-10

**Authors:** Peter Mortensen, Anders I. Larsen, Mette K. Zebis, Mogens T. Pedersen, Gisela Sjøgaard, Lars L. Andersen

**Affiliations:** ^1^Novozymes A/S, Medical Centre, 2880 Bagsværd, Denmark; ^2^Arthroscopic Centre Amager & Gait Analysis Laboratory, Copenhagen University Hospital, 2650 Hvidovre, Denmark; ^3^Department of Nutrition, Exercise and Sports, Faculty of Science, University of Copenhagen, 2100 Copenhagen, Denmark; ^4^Institute of Sports Science and Clinical Biomechanics, University of Southern Denmark, 5000 Odense, Denmark; ^5^National Research Centre for the Working Environment, 2100 Copenhagen, Denmark

## Abstract

*Objectives*. This study investigated long-term effects and implementation processes of workplace strength training for musculoskeletal disorders. *Methods*. 333 and 140 laboratory technicians from private and public sector companies, respectively, replied to a 3-year follow-up questionnaire subsequent to a 1-year randomized controlled trial (RCT) with high-intensity strength training for prevention and treatment of neck, shoulder, and arm pain. Being a natural experiment, the two participating companies implemented and modified the initial training program in different ways during the subsequent 2 years after the RCT. *Results*. At 3-year follow-up the pain reduction in neck, shoulder, elbow, and wrist achieved during the first year was largely maintained at both companies. However, the private sector company was rated significantly better than the public sector company in (1) training adherence, (2) training culture, that is, relatively more employees trained at the workplace and with colleagues, (3) self-reported health changes, and (4) prevention of neck and wrist pain development among initially pain-free employees. *Conclusions*. This natural experiment shows that strength training can be implemented successfully at different companies during working hours on a long-term basis with lasting effects on pain in neck, shoulder, and arm.

## 1. Background

Musculoskeletal pain and disorders are a major public health issue with a significant burden on health care systems and loss of work ability and productivity [1–5]. Neck and shoulder pains are among the most common musculoskeletal disorders with individual costs ranging from minor episodes of pain to severe and chronic disability [6, 7]. Arm and hand pain are less prevalent than neck and shoulder pain but still have major impact on sickness absence [5]. Pain in the neck, shoulder, and arm is related to physical work stressors as repetitive work, forceful exertions, static muscle contractions, awkward postures, and psychosocial factors [2, 8]. Among others, laboratory technicians—known to perform repetitive and monotonous arm/hand work tasks—show a high prevalence of such pains [9, 10].

Specific strength training reveals promising results in rehabilitation of neck and shoulder pain among office workers [11–14] where even a single set of strength training to failure 3 times a week provides moderate reductions of headache and neck pain [12, 15]. The rehabilitative effect of specific strength training on pain in the neck and shoulders has also been shown among laboratory technicians, where a 20-week, 1-hour a week intervention was undertaken during working hours [16–18]. Despite these promising results effective long-term implementation of strength training at the workplace and during working hours remains challenging.

A major reason for not adhering to physical activity is “lack of time” [19] and this factor has to be taken into account even when the physical exercise is undertaken during working hours in training facilities located within meters from the work station [16, 20]. Even though training with colleagues at work can be a motivating factor [20], a randomized cross-over study of women doing group gymnastics for 45 min once a week did not show any reduction in neck pain [21]. Effective neck pain reducing strength training during working hours seems flexible regarding time-wise distribution of training sessions [22]. However, training at least one to two times per week [23] and with a certain intensity seems important regarding long-term pain reduction [24]. Waling and coworkers found strength, muscular endurance, and coordination training equally effective in reducing work-related neck pain [25], but, in a three-year perspective, no difference could be seen in comparison with a reference group and almost half of the subjects had persisting pain after three years [26]. Also, other studies have documented active training to have a neck pain reducing effect, but the positive results have been short-lived [27, 28]. Therefore, further studying the implementation and motivational processes regarding strength training at work on a long-term basis is relevant.

## 2. Aim

This study is a long-term (3-year) follow-up of a randomized controlled trial lasting for 1 year [16, 18]. After the controlled study was finished, the two participating workplaces (a private and public sector company) chose to implement and modify the initial training program on their own without direct guidance from the researchers. However, at the private sector company, one of the former researcher assistants was employed as training instructor (first author of this paper) and at the public sector company the exercises from the controlled trial—although with slight variations—were used. This 3-year follow-up study was planned only a few months before the 3-year follow-up which allowed us in a natural experiment to study the implementation process and effects on musculoskeletal pain and general well-being in a long-term perspective without influence of the researchers. Both companies had good experiences with the training and we therefore wanted to investigate whether the two participating companies also were able to maintain the positive effects achieved during the first year (i.e., the controlled study) regarding musculoskeletal pain (neck, shoulder, elbow, and wrist), training adherence, and other self-reported health parameters.

## 3. Methods

### 3.1. Study Design

The initial design—which this study builds upon—was a cluster randomized controlled trial in Copenhagen, Denmark, from January 2009 to January 2010 involving 537 laboratory technicians (85/15% women/men, mean age 42 (SD 10) years). The short-term effects (up to one year) on pain and work disability have previously been reported [16–18]. All employed laboratory technicians from two large industrial production units from the public and private sector were invited to participate. At both companies, the work involved repetitive tasks, such as pipetting and preparing vial samples for analysis, often multiple samples in large series. Seated work in awkward postures (microscopy and LAF bench work), and data processing on a computer including mouse work, that is, tasks that require precision in work and may result in extended periods of time spent in static working postures. Forceful exertions occur when lifting heavy flasks and containers with chemicals. Further, many work tasks in the laboratory are performed during time pressure.

Pain and disability were not specific inclusion criteria. Including all employees allowed us to study the effect of the intervention at the company level, as well as the treatment and preventive effect, respectively, among subgroups with and without pain at baseline.

In 2009, we sent an internet-based questionnaire to 854 prospective participants of which 669 replied. 104 declined to participate or did not reply to the question concerning participation. Exclusion criteria—which led to exclusion of 28 participants—were pregnancy and serious health conditions such as previous trauma or injuries, life-threatening diseases, and cardiovascular disease. Thereby 537 participants were included in the study and randomly assigned at the cluster level to training (*n* = 282 in 30 clusters) or control (*n* = 255 in 27 clusters). At baseline pain registrations were obtained from 361 and 168 participants from the private and public sector companies, respectively, due to 8 missing replies. The controlled study ended in January 2010, and with permission from the ethical committee to extend the study period we send an email-based follow-up questionnaire to the original participants in January 2012, that is, two years after the 1-year controlled study had ended. At 3-year follow-up 333 and 140 participants from the private and public sector companies, respectively, completed the questionnaire. During the controlled study (i.e., first year) all participants received training (cross-over design) [18]. Consequently, there was no control group at 3-year follow-up.

All participants gave their written informed consent to participate in this study, which conformed to the Declaration of Helsinki and was approved by the local ethical committee (HC2008103). This was registered with ClinicalTrials.gov (NCT01071980).

As mentioned earlier a former research assistant from the controlled study was employed as training instructor at the private sector company (author PM of this paper). The National Research Centre for the Working Environment provided guidelines for the public sector company. Hence, the 3-year follow-up study was not planned until a few months beforehand; thus, the expectation of follow-up questionnaires was no longer a motivation to continue training for the employees. The National Research Centre for the Working Environment sent out questionnaires and performed all data analysis, hence avoiding conflicts of interests. The training instructor from the private sector company is the first author and can therefore have conflicts of interests in the interpretation of the results. Therefore, the entire research team ensured a thorough revision of the paper before submission.

### 3.2. Implementation after the Controlled Study

After the 1-year randomized controlled trial the two participating companies chose to continue strength training of laboratory technicians during working hours. Based on correspondence with the two companies the implementation process and training programs are described as follows.

### 3.3. Private Sector Company

**Figure 1:**
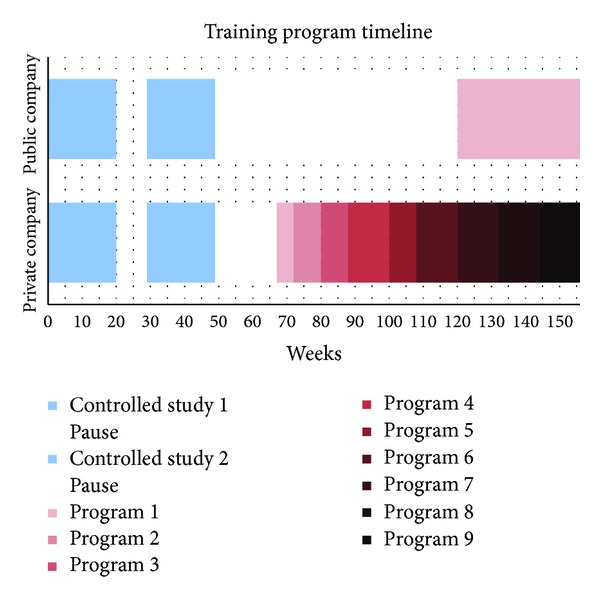
It shows the time course of training in the public and private sector companies, respectively. Blue colors define the controlled study period and red the natural experiment. The white blocks are periods where no guided training was offered at the companies. The controlled study was separated in two 20-week training blocks: first block with a training and control group and second block where the control group was offered the same intervention (cross-over design). The initial training group was allowed to carry on training during the whole controlled study period. After the controlled study the public sector company had a long break before training was restarted using the initial training program in a slightly modified version. At the private sector company nine different training programs were used throughout the natural experiment as described in Section 3. Hence, program 1 differed at the two companies despite the same coloring in the figure (see text for further explanation).

After termination of the controlled study the company employed one of the former researcher assistants as training instructor (sports physiologist and physiotherapist) for 24 hours a week to continue the strength training. First, a new strength training program was introduced in June 2010 (approximately 4 months after cessation of the controlled study—Figure 1) and nine different training programs with different exercises were introduced until February 2012 (programs 1–9). Each program lasted for 8–12 weeks (except program 1 lasting for 5 weeks—Figure 1). The initial seven programs consisted of 3 dumbbell exercises each* (program 1: one arm bent-over rowing, hammer curl, and incline front raise; program 2: one arm bent-over rowing, biceps curl, and shrugs; program 3: seated one arm front raise with opposite leg lift, side bend with shrug, and bent-over drag curl; program 4: lateral raise, seated back extension with row, and angled side bridge (dynamic); program 5: one arm curl and shoulder press, bent-over rowing, and incline push-ups; program 6: bent-over one arm rear lateral raise, front raise with back against wall, and reverse curl; program 7: Romanian deadlift with curl and shoulder press, gripper crushing, and palloff press (with elastic band))* and the last two programs contained six elastic band exercises each* (program 8: standing rear delt row, standing row, standing shoulder extension, lateral raise, front raise, and shoulder press—1st set full range of motion and 2nd set partials at end range of motion; program 9: horizontal shrugs with elastic band stretched between hands, overhead shrugs with elastic band stretched between hands, bent-over one arm shoulder flexion, standing torso twist, front raise with horizontal extension, and standing triceps extension)* (most exercises can be found in the following reference; others are self-invented combinations) [29]. Each program worked primarily neck, shoulder, arm, and back muscles, but abs were also engaged regularly.

All programs consisted of three training sessions each week containing 1-2 sets of 10–20 repetition maximum (RM) after a lighter warm up set. Trainees were instructed to gradually increase load over a few weeks until the given repetition number could barely be completed with proper form. Programs 1–4 had two exercises in each training session (alternating the three exercises for a total of 18 sets/week), programs 5–7 with all three exercises in each session (a total of 18 sets/week), and programs 8 and 9 with all six exercises in each session (a total of 36 sets/week). The circuit training principle was used in each program, keeping training intensity high with a minimal pause between sets. Hence most training session lasted for 5–10 minutes.

Programs 1–4 used a combination of linear and undulating periodization whereas programs 5–9 were not periodized. Progressive overload was conducted by constantly adjusting load to fit the given RM number. However, all subjects were carefully instructed by the training instructor to increase load only when all repetitions could be done without pain and with proper technique. In general repetitions were done with moderate pace. Training diary was part of the program but optional to fill out.

All nine training programs in the follow-up period were introduced by the training instructor to employees in classes of 5–15 persons for 30 minutes each. Classes were scheduled by the training instructor in collaboration with department managers and employees volunteered to participate. Program introductions took place in small training rooms (9 altogether—equipped with dumbbells, elastic bands, exercise pictures, and access to music) no more than 1-2 minutes of walk from the work station. Exercises were adjusted individually by the training instructor when needed. In the later programs instruction was guided by music to increase motivation. Training sessions besides the program introductions were nonsupervised. However, instructor/physiotherapist could be contacted by phone or mail in case of pain or other issues. Nonsupervised training was done alone or with colleagues in the above-mentioned training rooms.

Employees who were unable to attend the regular training sessions without pain or discomfort were offered a physiotherapeutic examination followed by a tailored personal training program.

### 3.4. Public Sector Company

After termination of the controlled study, training at the workplace was ceased for approximately 16 months while waiting for the research report with the main results. After the positive results from the controlled study were reported the company decided to restart the training. This was 9 months prior to the 3-year follow-up questionnaire (Figure 1).

Employees were introduced to the training program by the company's internal physiotherapist who was engaged with the training for approximately 1 day per week. Minor adjustments were introduced in the training program: dumbbells were replaced with elastic bands and one exercise was replaced following general training guidelines from the National Research Centre for the Working Environment. The 4 exercises were lateral raise (front raise as a variation), horizontal extension with elastic band between hands (reverse fly), external shoulder rotation from standing position with upper arm along torso, and wrist extension. The training program consisted of 1 set of maximum 20 repetitions in each exercise 3 times a week. Load was adjusted to fatigue the trainees during the 20 repetitions and when 20 repetitions could be performed with ease and proper form the load was increased [30]. With this method the load ranged between 10–25 RM. No pain was allowed during training and individual adjustments were introduced by the physiotherapist if needed. Trainees were advised to warm up with arm swings.

The program introduction lasted for 1 hour and afterwards 2 more follow-up sessions were given with 1-2-month interval to preserve proper technique. After this, supervision was offered approximately 4 times a year. Training videos with the 4 exercises were also available from the National Research Centre for the Working Environment [30]. Training introductions and follow-up supervisions were booked by interested employees and undertaken at department meetings. Regular training was performed alone or with colleagues in suitable places near the work station.

Employees with pain or other specific needs were offered an individual consultation with the physiotherapist, but no personal training program was offered.

### 3.5. 3-Year Follow-Up Questionnaire

In the randomized controlled study, participants replied to questions on pain and disability at baseline, after 20 weeks [16, 17] and after 1 year [18]. At 3-year follow-up, that is, two years after termination of the controlled study, participants received the same questions. Pain in neck, right shoulder, right elbow, and right wrist during the last three months was assessed on a 10-point scale from “no pain” to “worst imaginable pain.” Body regions were defined by drawings from the Nordic questionnaire [31]. Participants also replied to questions regarding training quality* ((1) level of effort during training, (2) most used training equipment, (3) training adherence: “How frequently have you trained your neck/shoulders/arms within the last year?,” *and* (4) training culture: (a) “Do you primarily train with colleagues, alone or both?” and (b) “Do you primarily train at work, at home/fitness center or both?”)* and self-reported health changes over the last year were included also* (question: “Which changes did you experience during the last year?” Reply categories were “Decreased,” “unchanged,” and “increased” in the following categories: “Wellbeing,” “musculoskeletal pain,” “muscle strength,” “physical reserves in daily life,” “mental reserves in daily life,” “social relations with colleagues,” “work enjoyment,” “desire to exercise,” and “desire to eat healthier”).*

### 3.6. Statistics

We used Proc Mixed of SAS controlled for gender and baseline pain to determine changes in pain between the two participating companies during the controlled study and natural experiment, respectively. We added a random cluster effect to the model to account for possible intracluster correlations. We did not impute missing data as all methods of data imputation have limitations [32]. Instead, the mixed procedure inherently accounts for missing data. We used the SAS statistical software version 9.2 for the analyses (SAS institute, Cary, NC) and accepted an alpha level of 5% as statistically significant. We report baseline results as means (SD) and changes from baseline to follow-up as means (95% confidence intervals (CI)) unless otherwise stated. Regarding the categorical data (adherence, health changes during the last year, training equipment, etc.) we used chi-square statistics to determine differences between the two participating companies and accepted an alpha level of 5% as statistically significant.

## 4. Results

### 4.1. First Year of the Intervention: The Controlled Study

**Figure 2:**
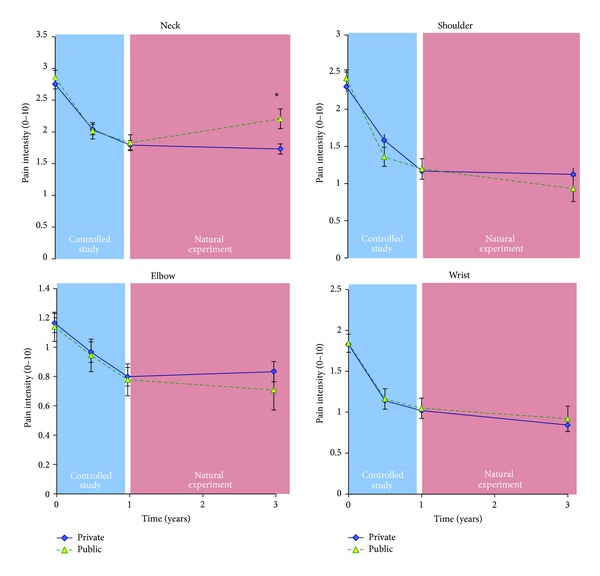
Pain among all participants from the two companies. Overall, the positive effects obtained after the first year were maintained at 3-year follow-up, except a tendency for neck pain increment in the public sector company. There was a main effect of time (*P* < 0.001) in all four regions during the controlled study (i.e., pain decreased), but not significantly during the natural experiment (i.e., the pain reduction was maintained). However, there was a borderline significant company by time interaction for neck pain, with a tendency for a worsening in the public sector company compared with the private sector company. *denotes a borderline significant company by time interaction for neck pain (*P* = 0.054) during the natural experiment.

Figure 2 shows the time-wise change in pain in the neck, shoulder, elbow, and wrist. During the first year, that is, the controlled intervention period, there was a significant decrease in pain in all four regions with no difference between the two participating companies (*P* value of* time* effect <0.001 in each of the four regions). In the private and public sector companies, respectively, pain intensity decreased significantly in the neck 0.93 (95% CI 0.71–1.14) and 0.80 (95% CI 0.46–1.15), in the shoulder 1.05 (95% CI 0.82–1.28) and 1.33 (95% CI 0.96–1.70), in the elbow 0.36 (95% CI 0.17–0.54) and 0.36 (95% CI 0.06–0.66), and in the wrist 0.81 (95% CI 0.59–1.02) and 0.72 (95% CI 0.37–1.07).

### 4.2. 3-Year Follow-Up: The Natural Experiment

*Pain.* Figure 2 shows for all participants that, from 1-year to 3-year follow-up, that is, during the two years after termination of the controlled study, there was a borderline significant* company by time* interaction for neck pain (*P* = 0.054). Post hoc tests showed a tendency for increased neck pain of 0.38 (95% CI −0.02–0.77) in the public sector company, but not in the private sector company. For the shoulder, elbow, and wrist there was no significant difference between the private and public sector companies, and there was no significant* time* effect, that is, the pain reduction achieved during the first year (the controlled study) was maintained during the two-year follow-up period (the natural experiment).

**Figure 3:**
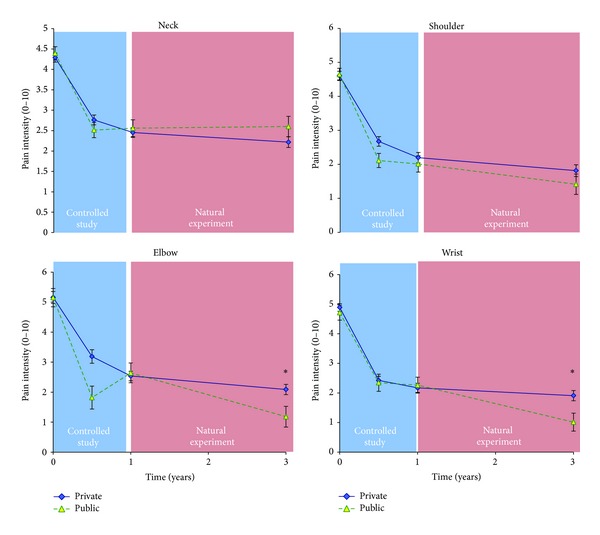
Pain among participants with pain (≥3) at baseline from the two companies. The positive effect obtained during the controlled study was maintained (private sector) or further decreased (public sector) during the natural experiment. *denotes company by time interaction for elbow pain (borderline, *P* = 0.054) and wrist pain (*P* = 0.04) during the natural experiment.

At baseline, the percentage with pain ≥3 in the neck, shoulder, elbow, and wrist was 51/53%, 42/46%, 19%/17%, and 30/35%, respectively, at the private/public sector companies.  Figure 3 shows the change in pain among those with pain (≥3) at baseline. The pain reduction achieved during the controlled intervention was either maintained or even further decreased. For the elbow there was a tendency (*P* = 0.054) and for the wrist a significant (*P* = 0.04)* company by time* interaction during the natural experiment, with the public sector company decreasing pain significantly more than the private sector company.

**Figure 4:**
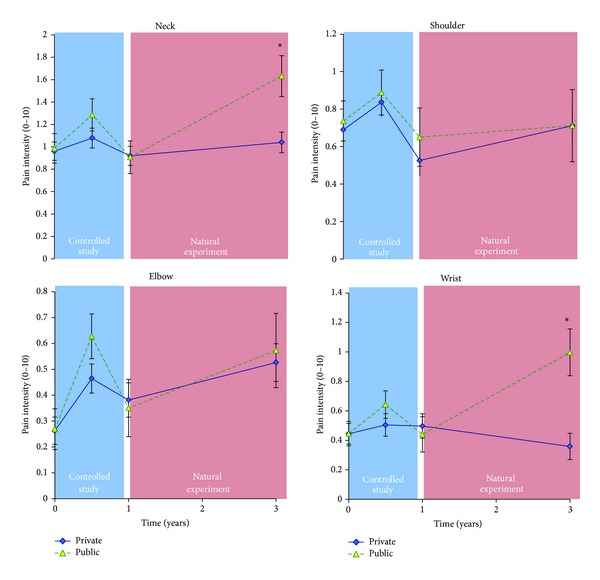
Pain among participants with no or little pain (0–2) at baseline from the two companies. Prevention of pain development was more effective for the neck and wrist in the private compared with the public sector company during the natural experiment. *denotes a significant company by time interaction for neck (*P* = 0.02) and wrist pain (*P* < 0.01) during the natural experiment.

Figure 4 shows the change in pain among those with no or little pain (0–2) at baseline. There was a significant* company by time* interaction for neck (*P* = 0.02) and wrist pain (*P* < 0.01), with the private sector company showing a significantly better preventive effect than the public sector company during the natural experiment.

### 4.3. Training Effort and Equipment

**Figure 5:**
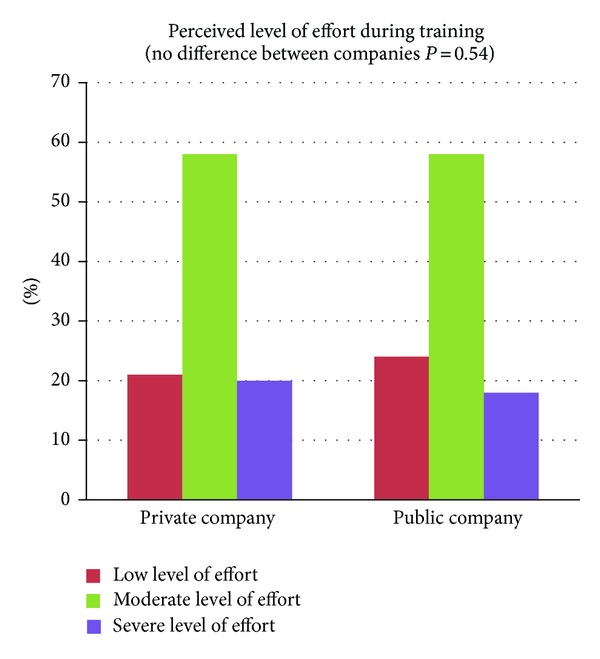
Perceived level of effort during training of neck, shoulder, and arm muscles throughout the natural experiment (private company: *n* = 333 and public company: *n* = 140). There was no significant difference in perceived level of effort between the two companies (*P* = 0.54).

Perceived level of effort during training of neck, shoulder, and arm muscles is shown in Figure 5. For both companies 21–24% reported low level of effort, 58% moderate level of effort, and 18–20% severe level of effort during training revealing no difference between the private and public sector companies (*P* = 0.54).

**Figure 6:**
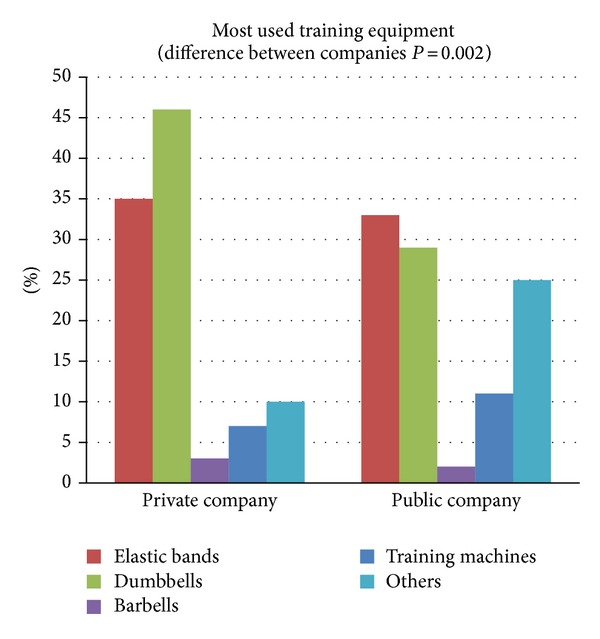
Most frequently used training equipment for neck, shoulder, and arm training throughout the natural experiment (private company: *n* = 333 and public company: *n* = 140). The two companies differed in the usage of training equipment (*P* = 0.002).

The most frequently used training equipment for neck, shoulder, and arm training was elastic bands and dumbbells (Figure 6). In the private sector company the training was carried out primarily with dumbbells and elastic bands; that is, 46% used dumbbells and 35% used elastic bands as training equipment most of the time. In the public sector company 33% and 29% of the employees reported elastic bands and dumbbells to be their first choice, respectively. Of the remaining population from the private and public sector companies, respectively, 3% and 2% used barbells, 7% and 11% used training machines, and 10% and 25% used other unknown equipment most of the time. There was a significant different usage of training equipment between the private and public sector companies (*P* = 0.002).

### 4.4. Training Adherence and Culture

**Figure 7:**
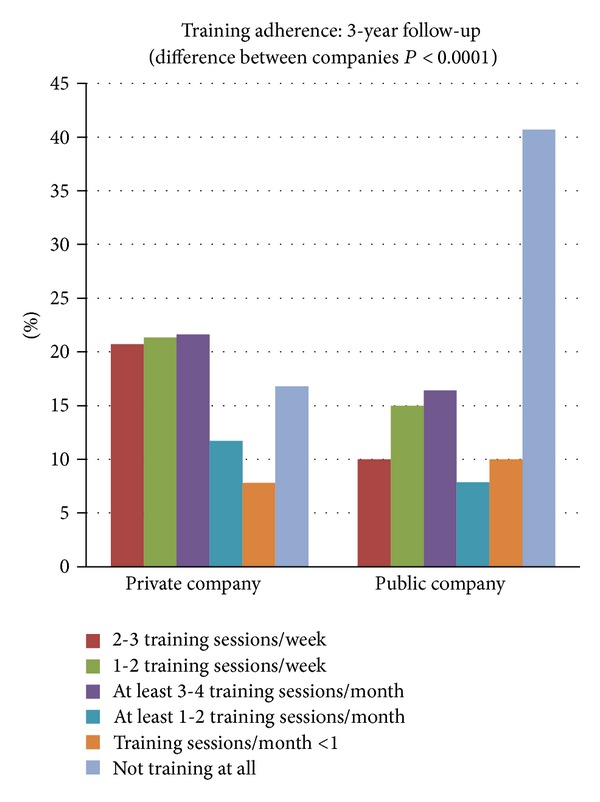
Training adherence during the last year of the natural experiment (private company: *n* = 333 and public company: *n* = 140). There was a significant difference in training adherence between the private and public sector companies (*P* < 0.0001).

Adherence was evaluated during the last year of the natural experiment (Figure 7). In the private and public sector companies 42% and 25%, respectively, participated regularly (i.e., at least once a week), 33% and 24% participated irregularly (i.e., between 1 and 4 times a month, but not every week), and 25% and 51% did not participate or participated less than once a month. The training adherence was significantly different between the private and public sector companies (*P* < 0.0001).

**Figure 8:**
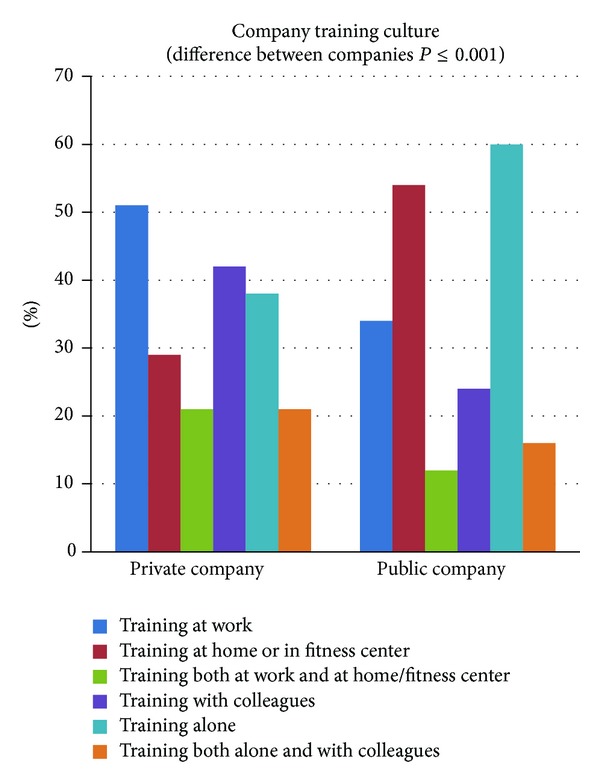
Illustration of the company training culture—where and with whom do employees train (private company: *n* = 333 and public company: *n* = 140). There was a significant difference in training culture between the two companies; training at home/fitness center or at work, *P* < 0.0001, and training with colleagues or alone, *P* = 0.001.

Figure 8 shows the distribution of where and with whom the employees trained. In the private sector company more employees trained primarily at the workplace (51%) compared to the public sector company (34%) whereas training at home or in a fitness center was more often performed among employees in the public sector company (54%) compared to the private sector company (29%). 21% in the private sector company and 12% in the public sector company trained both at work and at home/fitness center (significant difference between companies *P* < 0.0001).

In the private sector company significantly more employees trained primarily with colleagues (42%) compared to the public sector company (24%) (*P* = 0.001). Hence, more employees trained alone in the public sector company (60%) compared to the private sector company (38%) (*P* = 0.001). The remaining population trained equally alone and with colleagues, 21% and 16% in the private and public sector companies, respectively.

### 4.5. Self-Reported Health

Table 1 lists several self-reported changes in health, lifestyle, and work during the last year of the natural experiment among the employees. There was a significant difference between companies regarding wellbeing (*P* < 0.0001), musculoskeletal pain (*P* = 0.0005), muscle strength (*P* = 0.04), physical (*P* = 0.03) and mental reserves in daily life (*P* = 0.003), social relations with colleagues (*P* = 0.03), and work enjoyment (*P* = 0.005) favoring the private company. The desire to exercise (*P* = 0.39) and desire to eat healthier (*P* = 0.50) were not different between the private and public sector company.

## 5. Discussion

The main finding of this study is that the initial reduction of musculoskeletal pain after the 1-year randomized controlled trial was largely maintained at both companies after the natural experiment at 3-year follow-up.

Being a natural experiment the 3-year follow-up study did not allow us to have a control group without training, thus the interpretation of the results should be cautious. Also, differences in training programs and setup between the controlled study and the follow-up period and between the two companies blur rendering and comparison of results. However, the training setup in both companies originated from the same recommendations and experiences from the controlled study, and from the beginning of the controlled study, intention was to make the companies take ownership of the workplace training, enabling them to modify and implement training without guidance from the researchers after cessation of the controlled study. Therefore, this study is highly relevant and provides a detailed description of two different training setups helpful for companies or institutions aiming at improving the working environment.

Previous studies have documented active training to have a neck pain reducing effect, but the positive results have been short-lived [25–28]. Ylinen et al. have reported, maintained, and even improved effect on neck pain within one year of training [33]. Furthermore, Ylinen et al. showed the improvements from one year of intensive training to be maintained after 2 years further (3-year follow-up) [34]. A long-term (12 months) training induced pain reduction seems to be related to a high training intensity [24] and for both companies training intensity was high with 12 sets per week of 10–25 RM in the public company and 9 sets per week of approximately 10–20 RM + 9 lighter warm up sets in the private company. Training intensity and volume were comparable among the two companies, except for the last 20 weeks of the follow-up period where the private company did 18 sets + 18 warm up sets per week. When training is unsupervised actual training intensity is unknown but for both companies 21–24% reported low level of effort, 58% moderate level of effort, and 18–20% severe level of effort during training revealing no differences between companies (*P* = 0.54). These numbers indicate that not all employees trained with the intended 10–25 RM loadings. On the other hand, lifting to technical failure in exercises targeting small muscle groups like neck, shoulders, and arms can be done without experiencing severe level of effort. Optimally, training intensity could have been higher, but taking into account that training load was deliberately diminished while learning new exercises or due to pain and the fact that resistance training can be effective without the severe discomfort and acute physical effort associated with fatiguing contractions [35], training intensity in this natural experiment seemed acceptable.

At both companies training volume was decreased more or less equally compared to the initial training program in the controlled study [16]. The low-volume strategy with brief training sessions was chosen to keep training during working hours cost-effective and there is evidence that training quality was not compromised because clinically relevant reductions in pain and tenderness in neck and shoulders have been reported with only 3 weekly training bouts of a single set of approximately 20 repetitions to failure [12]. Furthermore, results from the controlled study revealed that even though training adherence was very high, the main reason for not attending training was lack of time [16, 20]; hence, it was assumed that brief training sessions would affect adherence and thereby long-term effect positively.

Myalgia of the neck/shoulder muscles is the most common clinical finding among people with neck/shoulder pain [36]. Neck/shoulder myalgia is characterized by muscular abnormalities such as reduced blood flow, increased anaerobic metabolism, increased muscle nociceptive substances [37, 38], inadequate capillary supply, and the presence of fibers bioenergetically deficient [39, 40]. Both strength and endurance training can elicit muscular adaptations reversing this process [41]. However, it is well established that significant morphological adaptations in muscles require months of intense training [42, 46], whereas gains in strength and muscle mass after 16 weeks of intensive strength training in untrained subjects could be maintained or even improved for months with one weekly training session in young adults [43]. The same pattern might be true regarding pain reduction where there is evidence that training frequency should be at least 1-2 times/week in the initial phase [14, 23], whereas improvements from one year of intensive training were maintained after further 2 years even though average training frequency was much lower during the follow-up period [34]. In the present study employees from both companies participated in at least 20 weeks of high-intensity strength training before the beginning of the follow-up period [18]. Thus, a long-term training effect was plausible even though training adherence was lower in the follow-up period compared to the first year of training. In contrast to the private sector company, the public sector company underwent approximately 16 months without workplace training after the controlled study, presumably affecting muscular development negatively. However, pain results at 3-year follow-up may be affected only to a minor extent due to the 9 months of training prior to the 3-year follow-up questionnaire. In support of this, studies reporting time-wise changes in pain have found the most marked pain reductions during the first four weeks of training [12]. Furthermore, in support of periodic training, Ogasawara and coworkers found that 3-week detraining/6-week retraining cycles elicited overall improvements in muscle size and strength similar to those occurring with continuous training after 24 weeks [44]. Andersen and coworkers found that pain reductions in response to 10-week high-intensity strength training in women with chronic neck pain were largely maintained during 10-week detraining [11]. As stated by Ylinen and coworkers and from experience in the private sector company, there is evidence that although several employees reported that they did not strengthen train regularly, they were going to recommence it if they felt symptoms in neck and shoulders [34]. Thus, some employees may have learned to manage their pain with intermittent training proposing a decent long-term training effect despite a low reported training adherence.

Limitations to the present findings and the findings of Ylinen et al. are the lack of a control group in both studies [34]. Hence, like the findings of Waling and coworkers, there is a chance that the long-term effect is affected only to a minor extent by the training interventions [26]. However, in the initial study of Waling et al. the training period was only 10 weeks (training equipment unavailable after this), the training had either too low intensity (endurance and coordination training) or was concentric training only (pneumatic resistance), and exercises were not sufficiently targeting the upper trapezius muscle being most prone to myalgia/trigger points [6, 45], which may explain the lack of long-term muscular adaptations and pain reduction [24, 42, 46–48]. These factors might also explain the modest effect measured immediately after the secession of the three different training regimes [26]. Other training studies on neck pain failing to show an effect [49] or long-term effect [27] share the above-mentioned limitations.

Training exercises that target specific muscles appear to be most effective in reducing neck/shoulder pain. The exercises of the controlled study revealed high muscle activity and specificity of the neck/shoulder muscles [50] and they proved to be effective in reducing neck and shoulder pain [16]. In the public sector company the exercises were quite similar to the original exercises. Shoulder external rotation was added which could be favorable because the primary external rotator of the shoulder is infraspinatus, which is next after upper trapezius most prone to painful muscular trigger points, and trigger points in infraspinatus are the major cause of unspecific shoulder pain [45]. Also, it should be mentioned that wrist extensor training was far more emphasized in this program compared to the private sector company training routines, which may explain the significant reduction of elbow and wrist pain in the public sector company during the natural experiment. The 4 exercises were done with elastic bands instead of dumbbells due to economical and practical reasons. Additionally, elastic bands are as effective as dumbbells in generating high levels of muscle activity [51]. At the private company approximately 30 different exercises for neck, shoulder, arm, and back muscles were used during the follow-up period although many exercises were close variations. This could be beneficial due to the fact that a broad variety of exercises and training regimes have shown to be effective in relieving neck and shoulder pain [13, 14, 24, 25, 27, 52–56] and variation in physiological stimulus can enhance continued progress by targeting more muscle fibers. Pain reduction can be achieved by training painful muscles [12, 16] but also by targeting nonpainful muscles within the same muscle synergy while avoiding intensive training of the painful muscles [57] which could further argue for a great variety in neck and shoulder exercises for rehabilitative purposes. Also, variation in exercises might be favorable in order to guide individuals tailoring a personal favorite program. In the private sector company the training was carried out primarily with dumbbells and elastic bands and the same was seen in the public sector company even though dumbbells were not accessible at the workplace, supporting that many employees trained either at home or in a fitness center. Data from the randomized controlled trial revealed the training program itself to be one of the key motivating factors for training adherence, but for most people lack of variation in training programs will make motivation fade over time. Lack of variation and absence of supervision might be key factors in the decreased training frequency seen in another 3-year follow-up study [34]. Therefore, the marked differences in exercises and program variations (including training diaries) among the private and public sector companies may underlie the significantly higher training adherence in the private sector company.

The higher training adherence in the private sector company might also be influenced by a better company training culture, that is, significantly more employees training at work and with colleagues in the private sector company compared to the public sector company. Training with colleagues can be challenging for laboratory technicians due to tight working schedules in the laboratory but this was equal for both companies. A good corporate training culture seems to depend on decent and easy accessible training facilities, getting support from company management, well-educated training instructors, but not the least socializing with colleagues during training [16, 20]. The private sector company had well-equipped training rooms (dumbbells, elastic bands, pictures of exercises, and music; anecdotally the latter seems to be important for having fun during training) within few minutes of walk from all work stations opposed to the public sector company having less equipment and more difficulty in finding suitable spaces to work out. Both companies had well-educated training instructors in comparable amounts of time: in the private sector company there was an average of 5 program introductions a year for a total of 2,5 hours* (277 training at private company; 10 persons in each class; total training time of 70 hours a year)* and at the public sector company they had 3 one-hour sessions introducing the program and afterwards supervision approximately 4 times a year* (83 persons training at public company; 10 persons average in each class; 25 hours for the program introduction and after this approximately 17 hours a year for supervision)*. It is noteworthy that hiring a trainer is an expense but little time is necessary to coach employees sufficiently even if new exercises have to be taught several times a year. Building a good corporate training culture takes time, favoring the private sector company having more training time throughout the natural experiment. However, it could be speculated that training culture and adherence could be affected negatively over time once the initial excitement is over especially if training routines are unaltered.

Differences in training programs, culture and increased adherence at the private sector company compared with the public sector company might account for the significantly better outcomes in self-reported wellbeing, musculoskeletal pain, muscle strength, physical and mental reserves in daily life, social relations with colleagues, and work enjoyment at the private sector company. As a consequence of training in the controlled study, most designated health parameters were improved for many subjects in both companies, but in the follow-up period most parameters were overall unchanged for the public sector company whereas the private sector company seemed to improve further. Only exception was the desire to exercise and eat healthier, where no difference was found between companies. This may in part be explained by these parameters being less related to training adherence. The present study cannot reveal whether the workplace strength training is accountable for these self-reported health changes at 3-year follow-up, but wellbeing, musculoskeletal pain, muscle strength, and physical and mental reserves in daily life can possibly be affected positively by strength training; hence, better training adherence and longer training duration during the natural experiment at the private sector company should be mentioned discussing these findings. Improved work enjoyment and social relations with colleagues are likely to be related to a social and fun training culture. Self-reported health changes during the last year are less valid and reliable than more objective measurements; however, they are important for the individual and for the employer as well.

The private sector company showed a significantly better preventive effect for neck and wrist pain than the public sector company among those with little or no pain at baseline (Figure 4). Among all participants there was also a borderline significant* company by time* interaction for neck pain favoring the private sector company (*P* = 0.054) (Figure 2). These findings may be attributed to high training adherence and long training duration. Among employees with pain at baseline (≥3), pain reduction achieved during the controlled study was either maintained or even further decreased (Figure 2). For the elbow there was a tendency (*P* = 0.054) and for the wrist a significant (*P* = 0.04)* company by time* interaction during the follow-up period, with the public sector company decreasing pain significantly more than the private sector company. Interpreting these findings it should be mentioned that the public sector company had some manual laboratory work automatized for 16 persons whereas work burden at the private sector company was unaltered with a tendency towards an increase. However, having no data supporting this, elucidation should be done cautiously. Another interpretation could be that the training program at the public sector company was more effective in reducing arm pain, focusing more on wrist extensor training. However, despite marked differences between the training programs of the two companies, it is impossible to conclude whether these differences in training stimuli can account for the findings of this natural experiment.

For the public sector company no data on severe work related musculoskeletal disorders have been available, but, based on company records at the private sector company, the reported number of disorders among laboratory technicians went from 7 in 2008 to 0 in 2012. In continuation, it is necessary to mention that around 100 persons had an individual examination and a tailored strength and stretching program from the physiotherapist/training instructor during the follow-up period due to musculoskeletal pain and disability. Most of these became pain free within few months of individual training. To our knowledge no serious adverse effects have been provoked by training in the follow-up period in both companies presumably due to proper instruction and individual adjustments in case of pain.

Specific strength training, on top of good ergonomic practice, can be a powerful tool in prevention and rehabilitation of pain and disability in neck, shoulder, and arms. However, the effect on a long-term basis relies on training adherence since training induced muscular adaptations diminish rapidly with detraining [42, 58–60], especially with increasing age [43]. However, this part is virtually impossible to test in a natural experiment as individuals developing pain are more likely to pick up training again than individuals without pain, creating a selection bias. Optimally, this should be tested in long-term randomized controlled trials. However, in a real-world setting it can be difficult for companies and individuals to commit and adhere to trials lasting for several years. Thus, natural experiments—as in the present study—may be the only practical way to study long-term adherence and effects of such interventions at companies.

## 6. Conclusion

In conclusion, this natural experiment at two large companies showed that strength training can be implemented successfully during working hours on a long-term basis with a lasting effect on pain in neck, shoulders, and arms. Key factors in training seem to be high intensity and specific exercise selection for neck, shoulder, and arm muscles.

## Figures and Tables

**Table 1:** Self-reported changes in physical and mental health and well-being during the past year. There were significant differences between companies in all parameters, except for the desire to exercise and eat healthier (see *P*-values in the table).

Self-reported health changes during the last year of the natural experiment		
	Private company %	Public company %
Wellbeing (difference between companies *P* < 0.0001)		
Decreased	5	11
Unchanged	65	75
Increased	30	14
Musculoskeletal pain (difference between companies *P* = 0.0005)		
Decreased	27	14
Unchanged	62	68
Increased	10	18
Muscle strength (difference between companies *P* = 0.04)		
Decreased	7	15
Unchanged	67	63
Increased	26	22
Physical reserves in daily life (difference between companies *P* = 0.03)		
Decreased	8	16
Unchanged	72	68
Increased	20	16
Mental reserves in daily life (difference between companies *P* = 0.003)		
Decreased	8	16
Unchanged	70	70
Increased	23	14
Social relations with colleagues (difference between companies *P* = 0.03)		
Decreased	5	9
Unchanged	80	81
Increased	15	9
Work enjoyment (difference between companies *P* = 0.005)		
Decreased	5	8
Unchanged	80	85
Increased	16	7
Desire to exercise (no difference between companies *P* = 0.39)		
Decreased	5	9
Unchanged	61	58
Increased	35	34
Desire to eat healthier (no difference between companies *P* = 0.50)		
Decreased	2	4
Unchanged	71	65
Increased	27	32
